# Invasion of the Toe-Nail

**Published:** 1857-03

**Authors:** 


Invasion of the Toe-Nail.—A new plan for the treatment of this painful affection is thus given by Mr. Lovegrove: "The nail, which is usually very thick on the great toe, was scraped moderately thin with a piece of glass, and then the whole surface covered with a good coating of nitrate of silver; which was accomplished by rubbing the stick of silver carefully over the whole nail, moistened with a little water, after which a linseed-meal poultice (hot) was applied, and the next morning nearly the whole of the nail was separated from the flesh, and another milder application divided it entirely. The nail was then removed without the least pain, and the patient assured me she had not suffered at all, during the whole operation. In less than a fortnight after the operation was completed, the patient wore her usual boots with comfort, and before leaving Brighton a new nail was rapidly growing."—*London Lancet.*

